# K^+^ binding and proton redistribution in the E_2_P state of the H^+^, K^+^-ATPase

**DOI:** 10.1038/s41598-018-30885-w

**Published:** 2018-08-24

**Authors:** Vikas Dubey, Minwoo Han, Wojciech Kopec, Ilia A. Solov’yov, Kazuhiro Abe, Himanshu Khandelia

**Affiliations:** 1 Department of Physics, Chemistry and Pharmacy, University of Southern Denmark, Odense, 5230 M Denmark; 2 MEMPHYS-Center for Biomembrane Physics, Odense, Denmark; 3 Computational Biomolecular Dynamics Group, Max Planck Institute for Biophysical Chemistry, 37077 Göttingen, Germany; 4 Cellular and Structural Physiology Institute and Department of Medicinal Science, Graduate School of Pharmaceutical Sciences, Nagoya University, Nagoya, 464-8601 Japan

## Abstract

The H^+^, K^+^-ATPase (HKA) uses ATP to pump protons into the gastric lumen against a million-fold proton concentration gradient while counter-transporting K^+^ from the lumen. The mechanism of release of a proton into a highly acidic stomach environment, and the subsequent binding of a K^+^ ion necessitates a network of protonable residues and dynamically changing protonation states in the cation binding pocket dominated by five acidic amino acid residues E343, E795, E820, D824, and D942. We perform molecular dynamics simulations of spontaneous K^+^ binding to all possible protonation combinations of the acidic amino acids and carry out free energy calculations to determine the optimal protonation state of the luminal-open E_2_P state of the pump which is ready to bind luminal K^+^. A dynamic pK_a_ correlation analysis reveals the likelihood of proton transfer events within the cation binding pocket. In agreement with *in-vitro* measurements, we find that E795 is likely to be protonated, and that E820 is at the center of the proton transfer network in the luminal-open E_2_P state. The acidic residues D942 and D824 are likely to remain protonated, and the proton redistribution occurs predominantly amongst the glutamate residues exposed to the lumen. The analysis also shows that a lower number of K^+^ ions bind at lower pH, modeled by a higher number of protons in the cation binding pocket, in agreement with the ‘transport stoichiometry variation’ hypothesis.

## Introduction

The gastric proton pump, H^+^, K^+^-ATPase (HKA) is a member of P_2_-type ATPase family, which is found in the stomach parietal cells. The HKA plays a crucial role in the acidification of the stomach via proton secretion and activates the digestive enzyme pepsin^1^, by mediating the electroneutral transport of protons into the lumen and potassium ions from the lumen against their concentration gradients using ATP as an energy source^2^. The pump maintains a proton concentration gradient of more than a million fold across the cell-membrane, which is the highest among P_2_-type ATPases. As a result, an astonishing pH ~ 1 is generated in the stomach lumen^3^.Like other P_2_-type ATPases, the *α* subunit of the HKA is responsible for the catalytic function of the enzyme and contains a cation binding domain and phosphorylation/dephosphorylation site. The ion exchange mechanism in the HKA and related ATPases is achieved through coupled phosphorylation and dephosphorylation reactions, commonly known as the Post-Albers reaction cycle^4,5^, which is based on two major conformation states, the cytoplasmic-open state (E_1_) and the luminal-open state (E_2_). In the E_1_ state, the HKA binds to protons from the cytoplasm, which triggers the formation of a phosphorylated intermediate E_1_P state via ATP hydrolysis followed by a conformational transition to the E_2_P state. In the E_2_ state, the protons are released in the stomach lumen, followed by binding of K^+^ ions^5–8^ from the stomach lumen. Occlusion of K^+^ in the E_2_P state dephosphorylates the enzyme and forms the K^+^-bound occluded state, which finally gets converted back to the E_1_ state by releasing bound K^+^ ions into the cytoplasm.

*In-vitro* biochemical measurements predict the transport stoichiometry to be 2 H^+^/2 K^+^/1ATP at neutral pH^9^. However, the energy generated from the consumption of one ATP is about −13 kcal/mol^10^, which is only sufficient to transport two protons against the maximum pH gradient of 4.7 units. Thus, it is thermodynamically not feasible to transport two cytoplasmic protons in exchange of two extracellular potassium ions *in-vivo* against the stomach pH gradient of 6 or more units^11^. Therefore, an alternative stoichiometry 1 H^+^/1 K^+^/1 ATP must operate under extreme acidic conditions to meet thermodynamic requirements^9,12^. Such pH-dependant variation in the binding stoichiometry was termed the “transport stoichiometry variation” hypothesis. One plausible conclusion that can be drawn from this hypothesis is that the pK_a_ of one acidic residue in the cation binding site is extremely low at pH ~ 1 and one of the two proton or potassium binding sites maintains a higher pK_a_, which allows acidic amino acid protonation under extreme acidic conditions. Structural evidence for the hypothesis emerged with a low-resolution cryo-EM structure where a single Rb^+^ was bound to the cation binding site in the E_2_P state of the HKA^11^.

**Figure 1:**
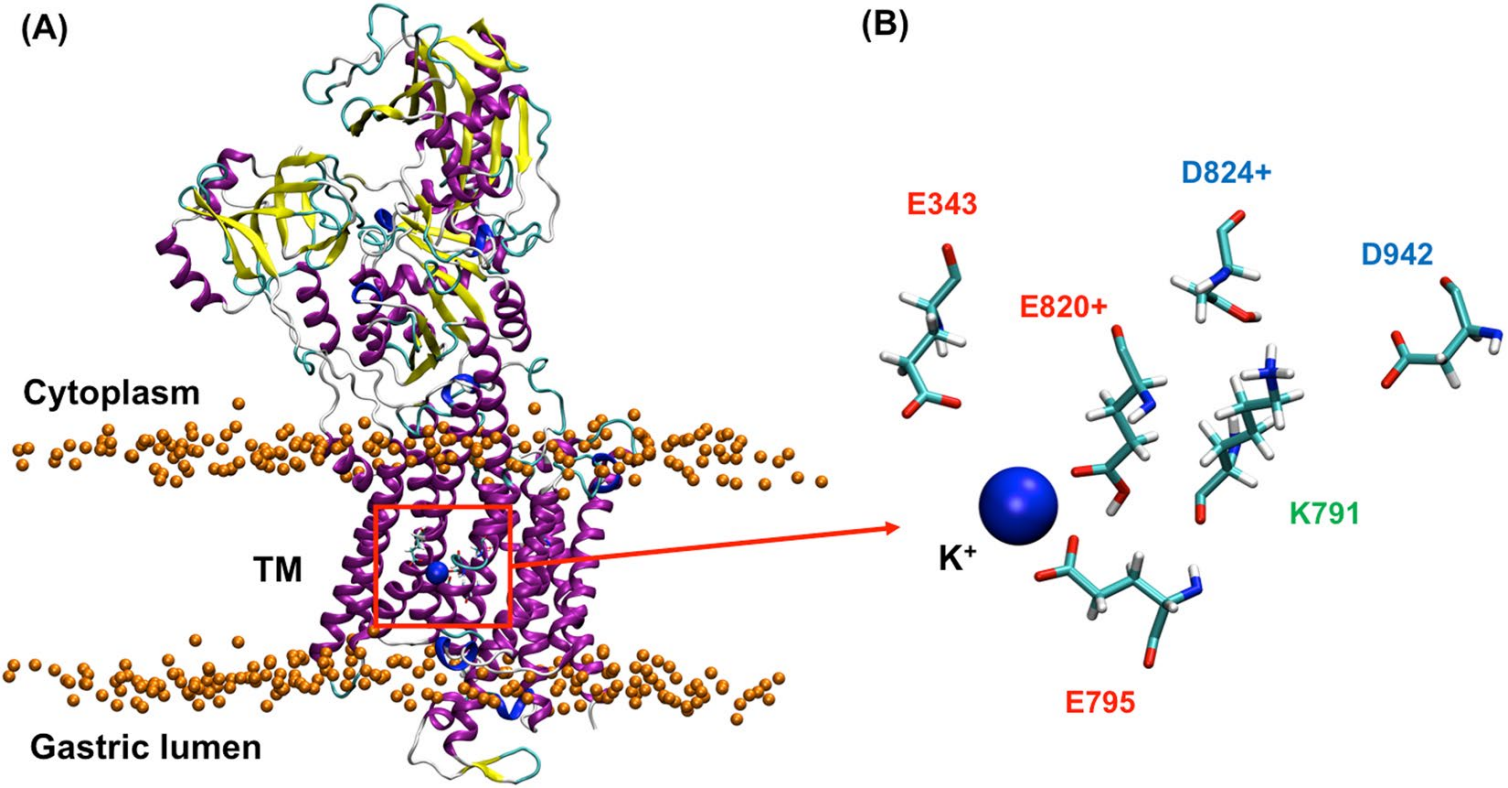
(**A**) The E_2_P-like H^+^, K^+^-ATPase model system embedded in POPC bilayer membrane. Water molecules and the lipid chains are omitted for clarity. Orange and blue beads represent the position of phosphorus atom in POPC lipids and bound K^+^ in the binding pocket, respectively. The red box shows the ion binding pocket (**B**) Five key acidic residues and one positively charged K791 residue in the cation binding pocket. E820 and D824 are protonated in this image.

The cation binding site of HKA is located in the middle of transmembrane (TM) region, which contains 10 TM helices (Fig. 1A) and 5 acidic residues: E343, E795, E820, D824 and D842 (Fig. 1B), which are found on TM4, TM5, TM6 and TM8^2,3^. Such an arrangement of carboxylic amino acids is similar to the cation binding sites of related P_2_-type ATPases: the Na^+^, K^+^-ATPase and the Ca^2+^-transporting SERCA^13^ pump. One key difference between the fundamental arrangement of the binding pocket of the HKA is the presence of the basic residue K791 on TM5 (Fig. 1), which replaces a serine in the Na^+^, K^+^-ATPase^2^. A cysteine to arginine mutation in TM8 of the normally electrogenic Na^+^, K^+^-ATPase converts it to an electroneutral pump like HKA^14^, highlighting the importance of a positively charged residue in the binding site of HKA. K791 along with E820 are regarded as crucial to proton transfer and the selective K^+^ binding in the E_2_P state of the HKA. Homology modeling predicts the presence of an E_2_-specific salt-bridge between E820 (TM6) and K791 (TM5)^15^, which has since shown to be critical in the operation of the pump^16–18^. The key idea is that the positively charged K791 residue acts as a Na^+^ or H^+^ surrogate and binds to Na^+^-binding site III in the E_1_.ATP state of the Na^+^, K^+^-ATPase. In the E_2_ or E_2_P state, K791 binds and stabilizes a negatively charged amino acid (probably E820) in the binding site in the absence of K^+^. The role of acidic residues in the cation binding site was extensively analyzed in a series of point-mutations^17–22^. The effect of charge-neutralizing mutations (sometimes in combination with mutations of other residues like K791) on the E_1_-E_2_ conformational equilibrium and the apparent K^+^ binding affinity via ATPase activity were measured. It is now established that the E820Q mutant had K^+^-independent constitutive dephosphorylation activity and an increased preference for the E_1_ conformation, leading to the conclusion that E820 is an important residue for K^+^ binding in the E_2_P state, because the charge neutralizing E820 to E820Q mutation mimics constitutive K^+^ binding^17,19^. The E820D mutant remains active. The E343Q mutation demonstrates reduced activity, while the E343A, E343L and E343V mutations had no activity^22^, indicating that protonation of E343 could be dynamic and important for the conformational equilibrium. E795Q remained as active as the WT enzyme, suggesting that E795 can be constitutively protonated.

The passage of protons through the HKA remains a fascinating phenomenon owing to the remarkable ability of the pump to release protons into a very low pH environment. Unlike alkali cations, protons have to either piggyback on water molecules or will bind to the acidic amino acids thus “protonating” specific amino acids in the cation binding pocket. Such proton redistribution in the cation binding site of the closely related Na^+^, K^+^-ATPase has been extensively studied recently^8,14,23–28^ because conformation-dependant protonation and deprotonation of acidic residues is deemed important for the release of Na^+^ from the E_2_P.3Na^+^ state, and is proposed to be critical in imparting cation selectivity (Na^+^ versus K^+^) to the Na^+^, K^+^-ATPase.

Previous molecular dynamics (MD) simulations studies of the HKA^13,29^, though insightful, primarily focused on the K791 salt bridge, and simulations are considered short by current standards, and thus insufficient to probe the detailed dynamics of the cation binding site. Here, we build a homology model for the outward facing, ready to receive K^+^, E_2_P state of the pump, based on the structure of the pig Na^+^, K^+^-ATPase. We further refine the model based on a recently-published 6.5 Å cryo-EM-resolved inhibitor-BYK99 bound density map^30^ of the HKA in the E_2_P state. We use the model to implement extensive MD simulations and free energy calculations to evaluate the cation binding stability in all the plausible various protonation states of the cation binding site of the HKA. The binding of K^+^ to the E_2_P triggers dephosphorylation and occlusion, and at the same time alters the local electrostatic environment of the binding pocket and thereby changes the pK_a_ of the acidic residues. In this way, K^+^ can induce proton rearrangement in the ion-binding pocket. Therefore, the protonation states of the acidic residue in the open state and of the occluded state are likely to be different. We provide a plausible mechanism for proton rearrangement amongst the acidic residues of the cation binding pocket based on a pK_a_ correlation analysis.

## Results

### K^+^ binding to different protonation states

In all the simulations, at least one K^+^ binds to the cation binding pocket of the HKA. The timescale of K^+^ binding ranges from t = 0 ns to t = 250 ns, depending on the protonation state. Sometimes, K^+^ binds during the 50 ns equilibration phase. When such binding occurs, the time of binding is set to 0 (t = 0). The last observed ion binding event occurred at 250 ns in the E343+E795+ (copy 2), E795+D820+D824+ (copy 1) simulations. Please refer to Supplementary Figure SI1 for traces of binding of K^+^ in all simulations. To investigate the number and distribution of bound K^+^ in the binding pocket, we calculate the average number of bound K^+^ for three 250 ns MD trajectories with different initial velocity distributions, for all different protonation states. In general, the number of deprotonated residues in the cation binding site will correlate to the number of bound K^+^, but this can be influenced by other moieties like water molecules and backbone carbonyl groups which can contribute coordinating oxygen atoms to the cation. Furthermore, the distribution of the protonated residues on acidic residues and water molecules can also matter, as has already been speculated for the Na^+^, K^+^-ATPase^23–26^.

**Figure 2:**
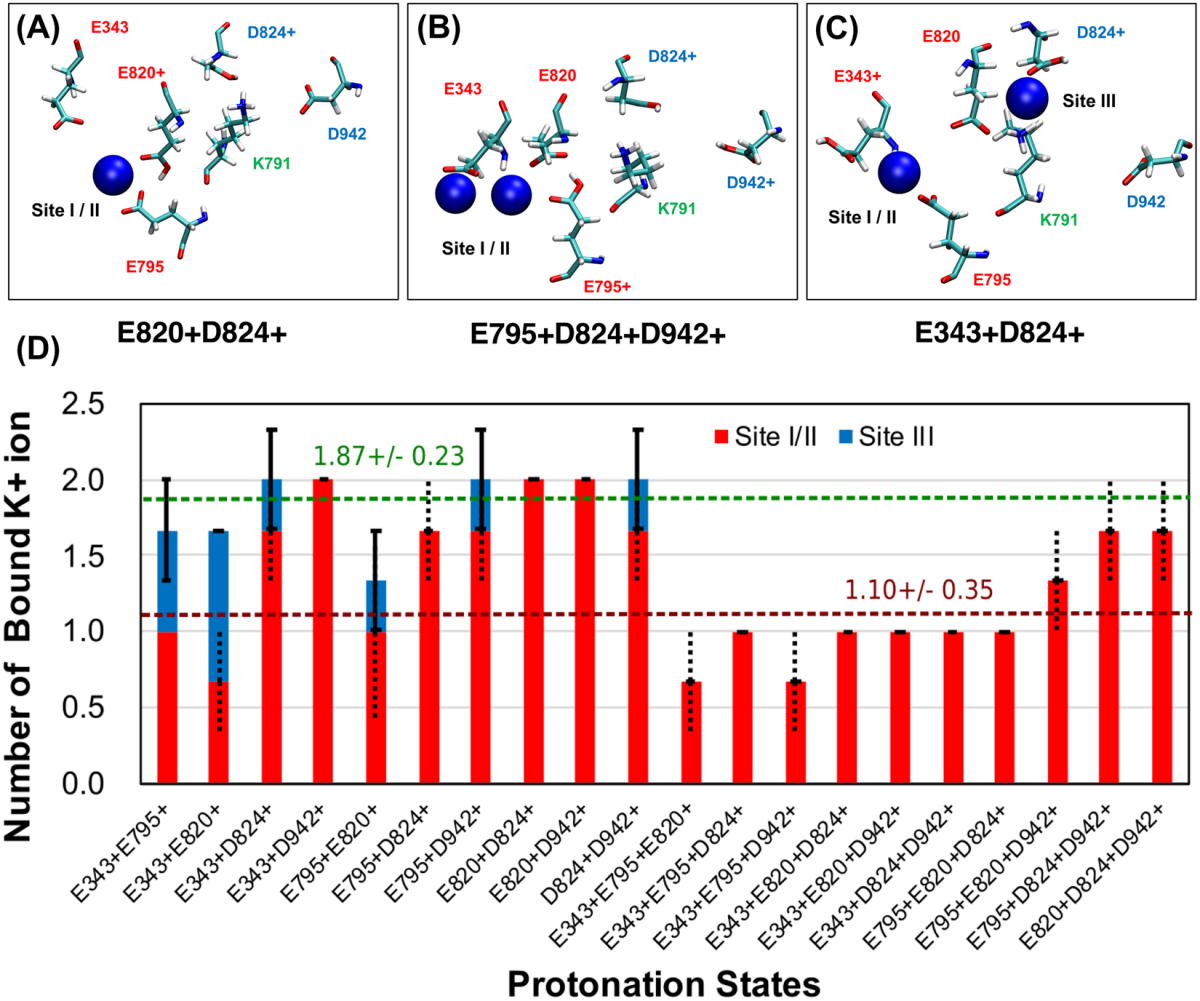
Three observed K^+^ binding conformations (**A**) 1 K^+^ is bound in site I/II (E820+D824+). (**B**) 2 K+ are bound in site I/II (E795+D824+D942+). (**C**) 1 K^+^ is bound in site I/II and another K+ is bound in site III (E343+D824+). (**D**) Average number of spontaneously bound K^+^ in 20 different protonation states with either 2 or 3 protonated residues in the binding pocket. Each bar represents the average number of bound K^+^ from three MD trajectories with different initial velocity distribution. The average number was calculated from the number of bound ions at the end of the each simulation. Red and blue bars represent the number of bound K^+^ ions in site I/II and site III, respectively. Dashed and solid lines on the bars show the standard error at site I/II and site III respectively. Sites I and II could not be distinguished in the simulations. Green and brown dashed horizontal lines show the averages of all the 2 protonated and 3 protonated states respectively.

Depending on the protonation state, different numbers of K^+^ ions bind to the cation binding site, and occupy different regions inside the binding pocket. Fig. 2A,B,C show three exemplary binding conformations of K^+^ and Fig. 2D shows the average number of bound K^+^ in all 20 considered protonation states. The *x-axis* in Fig. 2D shows the different protonation states and the number of bound K^+^ is averaged over three MD trajectories. The notation of each protonation state describes which residues are protonated amongst the five acidic residues in the cation binding pocket (Fig. 1B). For example, E343+E795+ refers to the simulation where the binding pocket has two protonated residues: E343 and E795, and the other residues, E820, D824 and D942, are deprotonated. We simulate all 10 protonation states possible with two protonated residues and all the 10 protonation states with three protonated residues. For the Na^+^, K^+^, ATPase, two K^+^-binding sites I and II were resolved in crystal structures^31,32^, and similar K^+^-binding sites were predicted for the closely related HKA^13^. However, at low stomach pH, the HKA will transport only one K^+^ ion. Cryo-EM structures with a single bound cation suggested that the K^+^-binding site close to E343 is likely to be a primary K^+^ binding site^11,33,34^ at low pH. This site was termed site II. In the simulations, however, a clear distinction between sites I and II could not be made. K^+^ binding is promiscuous and the K^+^ ions do not always remain bound to the same residues in the binding pocket. We use the term site I/II to indicate binding to both sites I and II. Figure 2A,B show that one or two K^+^ are bound in the site I/II. K^+^ ions bind to site I/II in all simulations where three residues are protonated (Fig. 2D). Interestingly, some simulations with two protonated residues show binding of K^+^ to site III (Fig. 2C). In particular, the E343+E795+ and E343+E820+ simulations show significant site III K^+^ coordination because both D824 and D942 are deprotonated. The average number of bound K^+^ in simulations with two and three protonated residues is 1.87 and 1.10 respectively over all 60 simulations, in good agreement with the stoichiometry variation hypothesis. In simulations with three protonated residues, there is an interesting negative correlation between the number of protonated glutamic acid residues and the number of K^+^ ions that bind to the cation binding pocket. Such a correlation is not detected for aspartic acid residues, and we attribute this to the vicinity of glutamate residues to the aqueous phase around the cation binding site I/II, compared to the aspartate residues, which are relatively embedded deeper in the cation binding pocket. Charge neutralizing mutations at E343 are known to reduce the K^+^ binding affinity^17^. In conventional MD simulation, protonated aspartate and glutamate residues will mimic a low pH environment. Thus, protonated glutamic acid residues in the luminal open E_2_P state represent a low pH condition in the gastric lumen. Simulations with three protons in the binding site represent a gastric lumen pH lower than that represented by simulations with two protons in the binding site. Thus, a state with fewer K^+^ bound with three protons represent a state equivalent to fewer K^+^ bound in a low pH environment which is in good agreement with “transport stochiometry variation” hypothesis.

The luminal open E_2_P state is more hydrated than the occluded state, and ion binding will be more diffuse than in the occluded state. Furthermore, an inhibitor like BYK99^30^ or SCH28080^11,33,34^, which bind to the luminal-facing conduit, can partially stabilize the dynamics in the cation binding site, and such an inhibitor is not present in the simulation models. Nevertheless, it cannot be ruled out that the low affinity binding observed in the simulations may be insufficient to trigger dephosphorylation and conformational change to the occluded state. It is also possible that actual proton rearrangement in the binding site after K^+^ binding stabilizes the binding pocket and also triggers occlusion. Such a possibility of explicit proton transfer cannot be modelled in classical MD simulations. However, we can deduce putative H^+^ redistribution within the cation binding site by carrying out a dynamic pK_a_ correlation analysis.

### Proton Redistribution upon K^+^ binding

**Figure 3:**
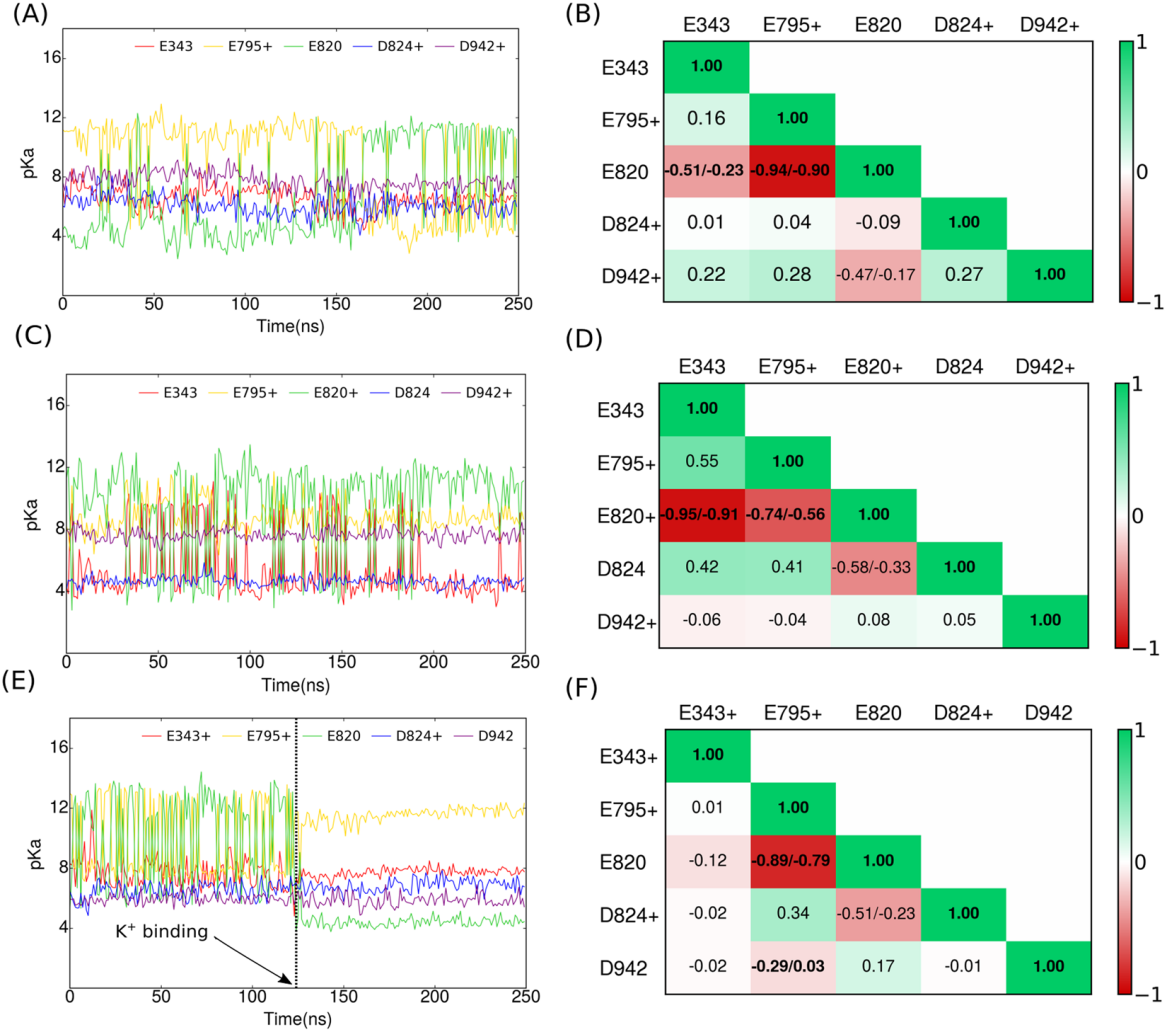
(**A**,**C**) and (**E**) pK_a_ fluctuations of the 5 key acidic residues in the cation binding pocket of the HKA during the 250 ns trajectories of the E795+D824+D942+, E795+E820+D942+ and E343+E795+D824+ simulations. (**B**,**D**) and (**F**) Pearson’s correlation coefficients (*r*) between pairs of acidic residues. The three highest values of *r* have been shown with 99% confidence intervals. The pK_a_ values are calculated every 1 ns. The red (*r* = −1) and green (*r* = 1) colours represent perfect negative and perfect positive linear correlation. The dashed line in (**E**) shows the time when K^+^ binds to the cation binding site (near t = 126 ns). In case of (**A**) and (**C**) K^+^ binds at the very beginning of the simulation (near t = 0 ns).

We calculate the pK_a_ of each acidic ion binding residue using PROPKA^35–37^, every 1 ns during the simulation. Note that H atoms are not included in pK_a_ using PROPKA. The pK_a_ values of the acidic residues do not only fluctuate around a constant value during the simulation (Fig. 3A,C,E), but change significantly because the environment of each residue changes dynamically. For example, the pK_a_ values of residues E795 and E820 in Fig. 3A change abruptly at 150 ns. Similarly, the pK_a_ of residues E795 and E820 change significantly upon binding of K^+^ at 125 ns (Fig. 3E). Thus, a single pK_a_ calculation based on a crystal structure may not always be useful inside a dynamic binding pocket. There is often a negative, and sometimes a positive correlation between the pK_a_ values for specific pairs of residues. For example, in Fig. 3A, E820 has a low pK_a_ and E795 has a high pK_a_ before 150 ns, while E820 has a high pK_a_ and E795 has a low pK_a_ after 150 ns.

To quantify the pK_a_ correlations, we calculate the standard Pearson’s correlation coefficient, *r* for each pair of acidic residues using:

1 $$r=\frac{\sum _{t=1}^{n}\,({x}_{t}-\bar{x})\,({y}_{t}-\bar{y})}{\sqrt{\sum _{t=1}^{n}\,{({x}_{t}-\bar{x})}^{2}}\sqrt{\sum _{t=1}^{n}\,{({y}_{t}-\bar{y})}^{2}}}$$

where *x*_t_ and *y*_t_ are the pK_a_ values of a pair of residues at time t and $\bar{x}$ and $\bar{y}$ are their average pK_a_ values over the last 100 ns of the trajectory. *n* is the number of snapshots for which the pK_a_ is calculated. *r* = 1 represents a perfect positive linear correlation, *r* = 0 is no linear correlation and *r* = −1 is perfect negative correlation.

There is a strong negative correlation between the pK_a_ values of E795 and E820 (−0.923) (Fig. 3B). We now make the following hypothesis: If there is a strong negative pK_a_ correlation between a pair of proximal residues only one of which is protonated, then it is probable that a proton can be transferred from the residue with a low pK_a_ to the residue with the high pKa. To compile the pK_a_ correlations, we generate a matrix for each simulation as shown in Fig. 3B,D,F.

**Figure 4:**
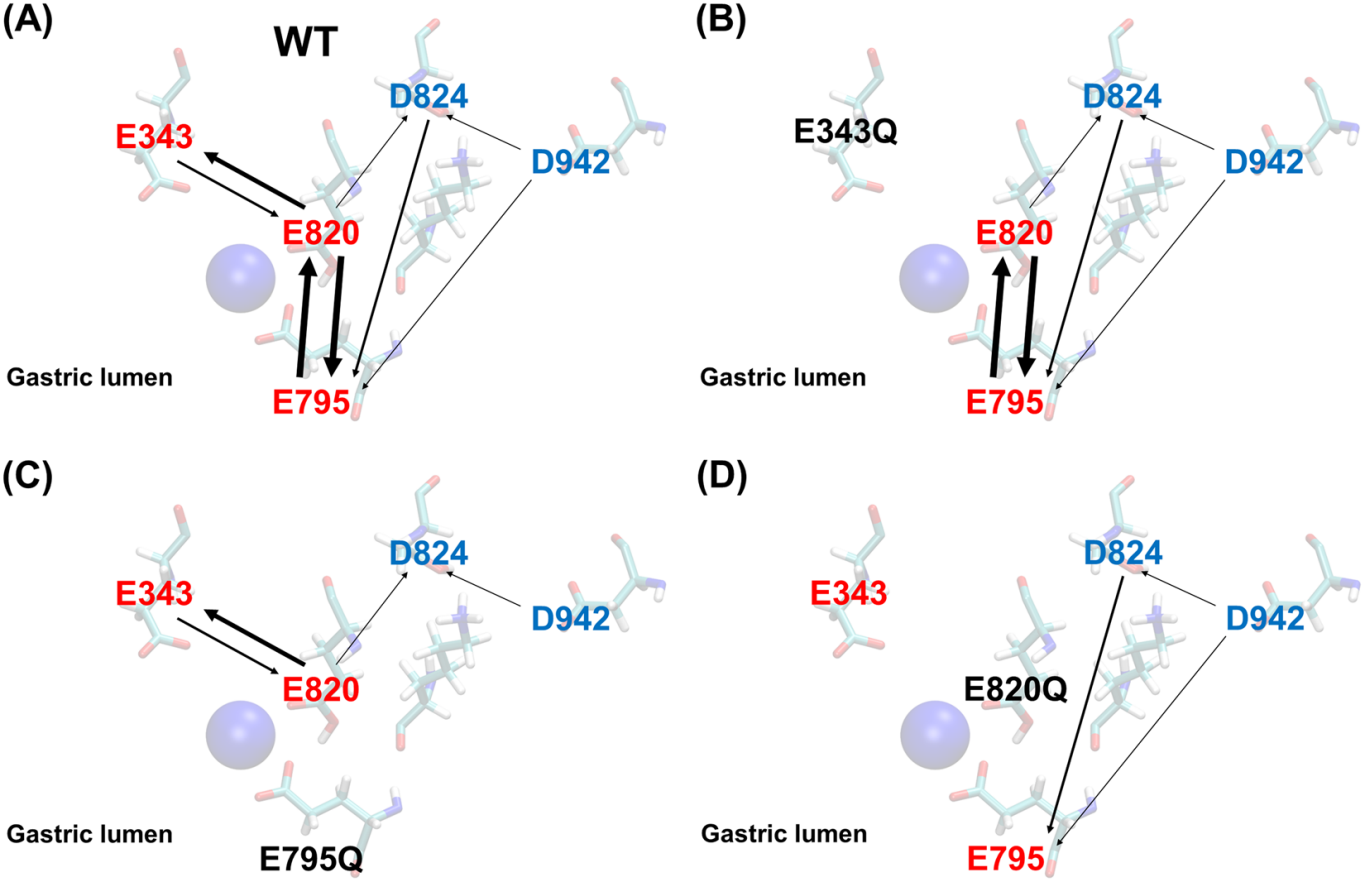
Diagrams of proton transfer pathways from pK_a_ correlation coefficient analysis in Table 1. The direction of the arrows represents the proton transfer direction and the thickness represent the likelihood of a proton transfer event calculated from pK_a_ correlation matrices. The transparent residues in the background are a guide to the eye, and do not represent a specific simulation.

After generating correlation matrices like the one shown in Fig. 3 for all 60 (20 protonation states, three copies of each simulation) trajectories, we select residue pairs, only one of which is protonated, and which have a strong negative pK_a_ correlation (*r* < −0.5). Table 1 enumerates all the pairs that have a strong negative correlation over the 60 trajectories. We can now make hypotheses about the likelihood of proton transfers between pairs of residues. The possibility of a proton transfer is constrained by simple physical requirements. First, a proton transfer is only possible if exactly one of the two residues is protonated. For example there is a strong negative correlation between E795+ and E820+ (Fig. 3C,D), but a proton cannot be transferred between the two because both are protonated. Secondly, the likelihood of a proton transfer depends also on the frequency (Table 1) with which negative correlations appear between a specific pair of residues over all 60 simulations. For example, a proton is more likely to transfer between E795 and E820 (5 instances of negative correlation), than between D942 and E795 (only one instance of negative correlation). Thirdly, the likelihood of a proton transfer between a pair residues is low if the residues are not proximal. Finally the depth of acidic amino acids from the external K^+^ access pathway indicates the possibility of a proton transfer into the bulk region. Based on these criteria, one can devise an ensemble of possible proton transfer pathways, as depicted in Fig. 4. Thus, one possible proton redistribution pathway is D824 → E795 → E820 or D824 → E795 → E820 → E343. Note, that for the WT enzyme, there is a very low likelihood that the proton will shuttle back into the binding site once it reaches E343 because the likelihood of a proton transfer from E820 to E343 is much higher than the reverse transfer. In protonation mimicking E → Q mutants (E343Q, E795Q and E820Q), the pathways for exchange of protons between the mutated residue and other residues are eliminated. Residue pairs predicted as more likely to exchange protons from pK_a_ correlations are also spatially close (Fig. SI2). We also constructed correlation matrices for systems analogous to those in Fig. 3, but with one less proton. Residues pairs predicted to exchange protons from the pK_a_ correlation analysis are identical for systems with 2 or 3 protons, please see Fig. SI3. Note that the information from Fig. 3 is not used to predict the ionization states of individual acidic amino acids, but to analyse correlations between pairs of amino acids to predict possible proton transfer events.

**Table 1:** The number of instances of each H^+^ donor and acceptor pair, which have a strong negative correlation coefficient of pK_a_ (*r* < −0.5) amongst the 20 different protonation states over all 60 simulations.

H^+^ donor	H^+^ acceptor	#instances
E795+	E820	5
E820+	E795	5
E820+	E343	4
E343+	E820	2
D824+	E795	2
E820+	D824	1
D942+	E795	1
D942+	D824	1

The pK_a_ is calculated from the 250 ns MD trajectories.

### Free energy calculations

**Figure 5:**
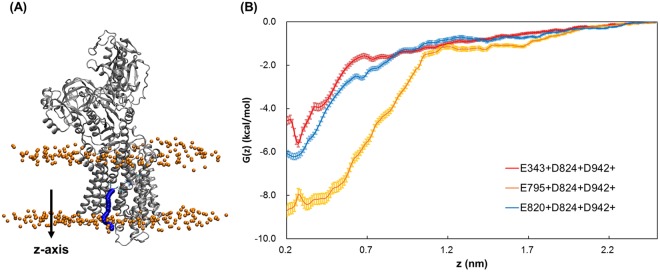
(**A**) The pulling pathway of the bound K^+^ ion from the binding site to the gastric lumen for the free energy calculations. The pathway is shown in blue and the arrow represents the pulling direction. (**B**) Potential of mean force along the *z-axis* computed for the three different protonation states as calculated with umbrella sampling. The error bars are obtained from bootstrapping analysis.

We carry out free energy calculations of K^+^ binding to the cation binding sites to determine the optimum protonation states for K^+^ binding. To limit the calculations to a reasonable number, D824 and D942 are always kept protonated. Deprotonation of D824 and D942 often leads to site III coordination (Fig. 2D), which is unexpected and not observed in cryo-EM structures. Furthermore, the pK_a_ analysis suggests that neither of D824 and D942 are as often involved in proton transfer as the glutamate residues, which have a strong tendency to redistribute protons amongst themselves (Table 1). We thus perform PMF calculations for three systems: where both D824 and D942 were always protonated, and one of the residues E343, E820 or E795 was protonated. The binding of K^+^ is strongest when E795 is kept protonated, followed by E820 and E343 (Fig. 5), suggesting that E795+D824+D942+ is the ideal protonation state which supports 1 H^+^/1 K^+^/ATP stoichiometry, and negatively-charged E343 and E820 are important for K^+^-binding. The differences in the binding energy from the PMF profiles correspond to an energy of about 5 *k*_*b*_*T*, and the theoretical precision of the potential of mean force calculations is 0.8 *kcal*/*mol* (equivalent to *k*_*b*_*T*).

**Figure 6:**
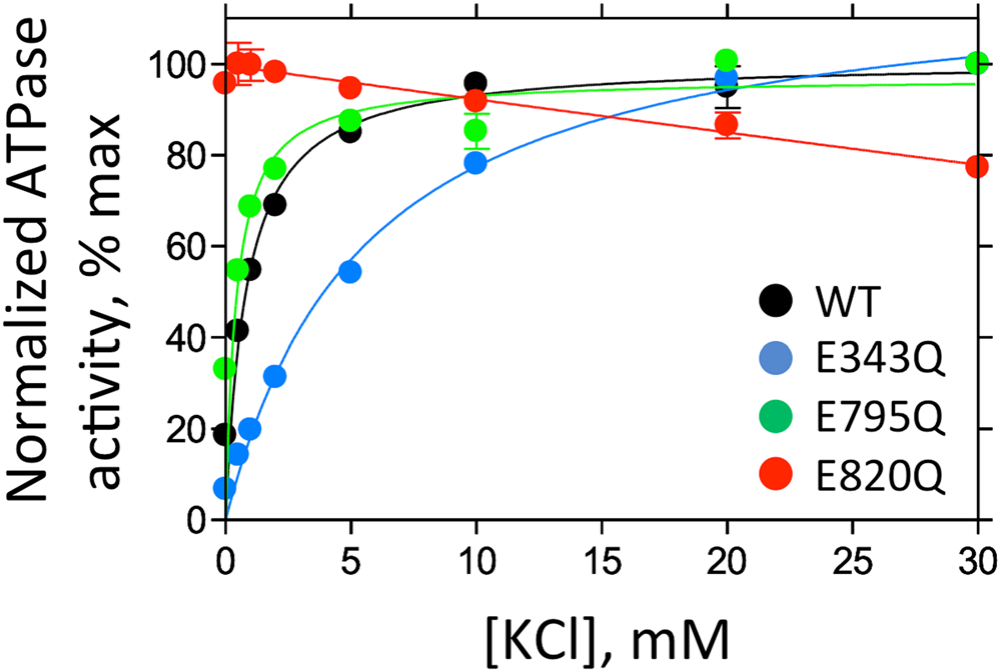
K^+^-dependence on ATPase activities of wild-type and indicated mutants. Data plotted were corrected for background values in the absence of K^+^ and in the presence of the specific inhibitor SCH28080, and normalized to their maximum velocity as 100% The affinities for K^+^ (K_m_) in our measurement showed 1.2 mM, 5.5 mM and 0.4 mM for wild-type, E343Q and E795Q respectively. The affinity for K^+^ of E820Q mutant could not be determined as this mutant showed K^+^-independent ATPase activity as reported previously^19^, see text for details.

The simulation results are in good agreement with prior measurements of apparent K^+^-binding affinities of various mutants^17–22^ which we have repeated and recompiled in the present work (Fig. 6). The E795Q mutation has no effect on the K^+^ affinity, suggesting that E795 is protonated, in agreement with the highest free energy of binding calculated from the PMF for the E795+D824+D942+ case. The binding free energy is halved when E343 is protonated, in good agreement with the reduction in the K^+^ binding affinity of this mutant. The E820Q mutant is constitutively active without K^+^ in the experiments. This K^+^-independent, constitutively active phenotype (E820Q) is interpreted as follows: the mutation mimics a charge neutralization of E820 by bound K^+^, thus E_2_P dephosphorylation is continuously induced^19^. Therefore, E820 is thought to be an important residue to sense the K^+^-binding, which in turn transmits the molecular signal to proceed with the transport cycle, consistent with the simulation result that deprotonated E820 is likely involved in the K^+^ binding. The proton redistribution pathways in Fig. 4 indicate that E820 acts as a key node for the redistribution of protons. Although speculative, this proton network may be related to the molecular signal that is induced by the K^+^ binding. Furthermore, we often observe K^+^ ions closely coordinated by E820 in several simulations, indicating that E820 is highly likely to bind a K^+^ ion after occlusion.

## Discussion

Amongst type II P-type ATPases, the dynamic protonation states of the cation binding acidic amino acids in the HKA pump are of particular interest, because the protein pumps protons against a millionfold concentration gradient. Our simulations show that the protonation states of acidic amino acids in the binding site and K^+^ binding are both highly dynamic in the water-filled cation binding cavity of the luminal open E_2_P state. We devise a simple pK_a_ correlation analysis that predicts the possible proton redistribution pathways in the cation binding site after the binding of K^+^, and carry out free energy calculations to compute the differences in the binding energy of K^+^ for the more likely protonation states in the binding pocket. We propose that residue E795 is protonated when the protein is ready to receive K^+^ ions under low pH conditions. The simulation data agrees well with previously published and new measurements of K^+^ binding affinity of charge-neutralizing E → Q mutations. During preparation of this manuscript, a 2.8 Å structure of the luminal open state was published^38^, in a complex with an inhibitor. The conformations of the acidic amino acids are in good agreement with those predicted in the current set of simulations. In particular, the proximity of E795 and E820 in the crystal structure strongly supports our hypothesis that these two residues can easily exchange a proton, which can subsequently be released into the lumen. The selectivity of K^+^ versus H^+^ in the luminal open state remains to be investigated, and will be addressed in the future free energy perturbation simulations using the newly available crystal structure. We find that the formation of the K791-E820 salt bridge has a complex dependance on the protonation state of not only E820, but also of other acidic residues in the binding pocket (data to be reported elsewhere). The conformations and interactions of K791 are likely to be closely coupled to the E_1_-E_2_ conformational transitions of HKA, and are outside the scope of the current work. In ongoing work, we are addressing the K791 facet of the HKA in simulations based on homology models of HKA built from high resolution crystal structures of the Na^+^, K^+^-ATPase in the E_1_.ADP.3Na^+^ state^39^. Finally, the analysis of protonation states and possible proton transfer events in other conformations of the pump remains to be investigated.

A key limitation of the current classical MD simulations is that there is no possibility to explicitly simulate proton transfer between chemical moieties such as amino acid residues and water molecules. All simulations here are based on a homology model, which can potentially diverge from the native state in very long (microseconds) simulations^40^. In the current case, however, the similarity of the structure, sequence and function of the HKA and the Na^+^, K^+^ ATPase, as well refinement of the homology model based on the cryo-EM density maps makes us relatively confident of the initial model. Agreement with the newly published crystal structure^38^ with respect to the overall binding site conformations is further evidence that the initial model is reasonable.

## Methods

### *In-vitro* measurement of HKA activity

Prior measurements of the *in-vitro* HKA activity and of its mutants were carried out for the rabbit enzyme^17,19–22^. Here, the wild-type (WT) and mutants of pig gastric *α*-subunit of HKA were co-expressed with WT *β*-subunit in HEK293S GnT1- cells, and broken membrane fractions were used for the ATPase activity measurement as described previously^30^. In our structure we used the pig enzyme purified from natural source, whose sequence is very similar (99%, only 14 aa difference) to the rabbit sequence, and there is no difference in the cation binding site. We aim to confirm that pig enzyme also showed similar phenotype compared with previously reported rabbit enzyme. In addition, our analysis with mammalian cell expression shows much higher (~2 micromol/mg/h) specific activities compared to previous sf9 expression (~0.1 micromol/mg/h), which gives a high S/N ratio for reliable results. Briefly, permeabilized membrane fractions were suspended in buffer comprising 40 mM PIPES/Tris (pH 7.0), 2 mM MgCl_2_, 2 mM ATP and 0–30 mM KCl, and incubated at 37 °C for 1 to 3 hours depending on their activity. Reactions were terminated, and the amount of released inorganic phosphate was determined colorimetrically. Data were plotted as specific inhibitor SCH288080-sensitive activity, and the maximum ATPase activity of indicated mutants were set as 100%. For the background, 10 *μ*M SCH28080 was added in the reaction system. Data were fitted by Michaelis-Menten equation using Graphpad Prism (version 7.00 for Windows, GraphPad Software, La Jolla California USA, www.graphpad.com).

### Construction of the HKA model system for MD simulations

The homology model of the pig HKA E_2_P state was built by threading the pig HKA sequence along the structure of the pig Na^+^, K^+^-ATPase, PDB id 4HYT^41^ (previously: 3B8E)^31^ using Modeller (v9.7)^42–45^. The sequence identity between the two proteins is 62.5% which is sufficiently large for a reliable homology model. The homology model was further refined using the 6.5 Å cryo-EM-resolved inhibitor-BYK99 bound density map^30^ of the HKA in the E_2_P state. Initially, the homology model was manually fitted into the density map, which was followed by adjustments for each individual domain and the TM helices within the EM density map using Situs^46^. After the positional search, further fine fitting and connecting the split loop region was performed manually using COOT with regularization refinement^30^. The $Al{F}_{4}^{-}$ group and the adenosine diphosphate (ADP) in the cryo-EM structured were not retained and D385 at the adenosine triphosphate (ATP) binding site was phosphorylated. For P-type ATPases, homology models have often made accurate predictions, that were confirmed in crystal structures solved later. Before the crystal structure of the Na^+^, K^+^-ATPase was deciphered, homology models (without MD simulations) based on the crystal structure of SERCA ATPase correctly predicted the ion-binding sites^47^. A homology model of the HKA based on the crystal structure of SERCA was also used to predict the hydrogen bond between K791 and E820^17^. Shorter (10 ns) MD simulations were also implemented for the HKA from homology models based on the SERCA pump^29^. The protein was embedded in a POPC bilayer, which was earlier used as an effective lipid matrix to analyze ion pumps^48,49^. Na^+^, Mg^+^ ions and water molecules from the initial structure were retained in the model. The POPC bilayer with 362 lipid molecules were constructed using CHARMM-GUI^50,51^ and hydrated with ~61,666 water molecules so the total system consisted of ~254,000 atoms including the HKA. The system was kept electroneutral with an addition of randomly distributed 13–14 K^+^ ions in the solution. The K^+^ binding pocket contains five acidic residues (Fig. 1). Simulations were run for 20 different possible protonation states when either two or three acidic residues of the cation binding pocket were kept protonated. Acidic residues not in the binding pocket were kept deprotonated. We do not consider protonation states which have 1, 4, or 5 protonated residues. When the number of protonated residues is 1, the protein conformations are unstable because of a significant net negative charge in the binding pocket. When the number of protonated residues is 4 or 5, there is a very low likelihood of K^+^ binding. Table S1 in the supporting information (SI) lists all 20 protonation states.

### Simulation details and data analysis

#### Molecular dynamics simulations

All-atom MD simulations were performed using GROMACS version 5.0.6^52–56^, with the CHARMM36 force field with CMAP correction^57–60^. The parameters for phosphorylated aspartate were taken from a previously developed parameter set^61^. We used the TIP3P water model with Lennard-Jones interactions on hydrogen atoms. A 12 Å cutoff was used for non-bonded neighbor list, and was updated every 10 integration steps. The van der Waals interactions were switched off between 10 to 12 Å. Electrostatic interactions were treated with the particle mesh Ewald (PME) method^62,63^. All systems were energy-minimized for 5,000 steps using the steepest descent algorithm, followed by a 50 ns equilibration and a subsequent 250 ns production simulation run. Three copies of each system were simulated, resulting in a total of 750 ns MD trajectories for each protonation state (see Table S1 in the SI for a list of all simulations). The temperature of the system was kept at 310 K with the Nosé-Hoover thermostat^64,65^ after an equilibration run which was performed with the Berendsen thermostat^66^. The pressure was kept at 1 bar with semi-isotropic pressure coupling realized with the Parrinello-Rahman barostat^67^ after equilibration with the Berendsen barostat^66^. The Linear Constraint Solver (LINCS)^68^ algorithm was used to constrain all bonds containing hydrogen. A 2 fs time step was used and trajectories were sampled every 50 ps. The data analysis was carried out using GROMACS and python scripts, while snapshots in figures were rendered using Visual Molecular Dynamics (VMD)^69^. All the pK_a_ values were estimated using the software PROPKA (v-3.1)^35–37^.

#### Binding free energy calculations

We used umbrella-sampling approach to calculate the 1-D potential of mean force (PMF) of a single K^+^ ion binding for selected protonated states to determine the optimal protonation state for K^+^ occlusion. Here, we assume that under optimal protonation conditions, the binding of a K^+^ ion would be energetically the most favourable (i.e. with the lowest Gibb’s free energy of binding). The last frame of the MD simulation, where a single K^+^ ion was initially bound to the binding site, was chosen as a reference structure and the carbonyl carbon of E820 was chosen as the reference atom for pulling. This was followed by the generation of windows based on the distance between the carbonyl carbon and only the bound K^+^ ion. The bound ion was pulled along the *z*-direction (bilayer normal) until it reached the water layer with a pull rate of 0.001 nm/ps for a time of 5 ns. A force-constant of 1000 *kJ*/*mol*.*nm*^2^ was applied on an ion during pulling. From the pulling simulation, a total number of 47 equidistant sampling windows was generated, covering the distance between 0.20 nm and 2.5 from the reference. The windows were separated by 0.05 nm. Subsequently, each window was equilibrated for 10 ns followed by a sampling simulation of 40 ns. A higher force-constant of 5000 *kJ*/*mol*.*nm*^2^ was applied to the bound ion to improve sampling in a given window. Additionally, the ion was restrained in the lateral direction by applying cylindrical flat-bottomed restraints along the z-axis with the cylinder radius = 1 nm, which is much larger than lateral fluctuations of the ions in the pathway leading to the binding sites. These restrains act on an ion only when it is trying to leave the cylinder, whereas the ion dynamics are not affected inside the cylinder. Such an approach has been widely used in PMF calculations^70,71^, as it avoids the problems that arise when the ion leaves the channel by effectively increasing the sampling in the unbound state. The rest of the simulation parameters were the same as in the MD simulations. Reweighing and PMF calculations were performed using the GROMACS WHAM tool^72^. Histogram overlaps are shown in Fig. SI4. 200 bins and 200 bootstraps were used to obtain average PMF values and error estimates. All the profiles were shifted so that the bulk PMF values were zero. In general, we used the same methodology as Hub *et al*.^72^ and Allen *et al*.^73^ and reviewed extensively by Deng and Roux^74^, which tends to generate accurate results. For example, Hub *et al*. observed good agreement between the permeability derived from free energy calculations and the experimental one in the case of the Rhesus protein channel^72^. Similarly, the dissociation constant computed from the PMF of the phosphate ion diffusing through the outer membrane channel OprP compares well with experiments^75^. Limited sampling in free energy calculations that can affect the accuracy of the final result. We are rather confident that 40 ns per window (2 microseconds per single PMF curve) is sufficient to obtain a converged PMF in a meaningful structural context, capturing the backbone and sidechains dynamics of the protein^76^.

No animal or human subjects were used in this study.

## Electronic supplementary material

Supplementary Information
