# Supplementary material for: K+ binding and proton redistribution in the E2P state of the H+, K+-ATPase
Source: Sci Rep. 2018 Aug 24;8:12732. doi: 10.1038/s41598-018-30885-w (PMC6109069; doi:10.1038/s41598-018-30885-w)

Supporting information for
K^+ binding and proton redistribution in the E_2P state of the H^+, K^+-ATPase

Vikas Dubey[1,2], Minwoo Han[1,2], Wojciech Kopec[3], Ilia A. Solov'yov[1], Kazuhiro Abe[4], and Himanshu Khandelia[1,2,*]

[1]Department of Physics, Chemistry and Pharmacy, University of Southern Denmark, Odense, Denmark 5230 M
[2]MEMPHYS-Center for Biomembrane Physics
[3]Computational Biomolecular Dynamics Group, Max Planck Institute for Biophysical Chemistry, 37077 Göttingen, Germany
[4]Cellular and Structural Physiology Institute and Department of Medicinal Science, Graduate School of Pharmaceutical Sciences, Nagoya University, Nagoya 464-8601, Japan
[*]hkhandel@sdu.dk

**2 Protonated Residues**	**3 protonated residues**
E343+E795+ **X 3**	E343+E795+E820+ **X 3**
E343+E820+ **X 3**	E343+E795+D824+ **X 3**
E343+D824+ **X 3**	E343+E795+D942+ **X 3**
E343+D942+ **X 3**	E343+E820+D824+ **X 3**
E795+E820+ **X 3**	E343+E820+D942+ **X 3**
E795+D824+ **X 3**	E343+D824+D942+ **X 3**
E795+D942+ **X 3**	E795+E820+D942+ **X 3**
E820+D824+ **X 3**	E795+E820+D824+ **X 3**
E820+D942+ **X 3**	E795+D824+D942+ **X 3**
D824+D942+ **X 3**	E820+D824+D942+ **X 3**

Table S1: List of the states for which MD simulations were performed. For each state, 3 copies of simulations have been performed.

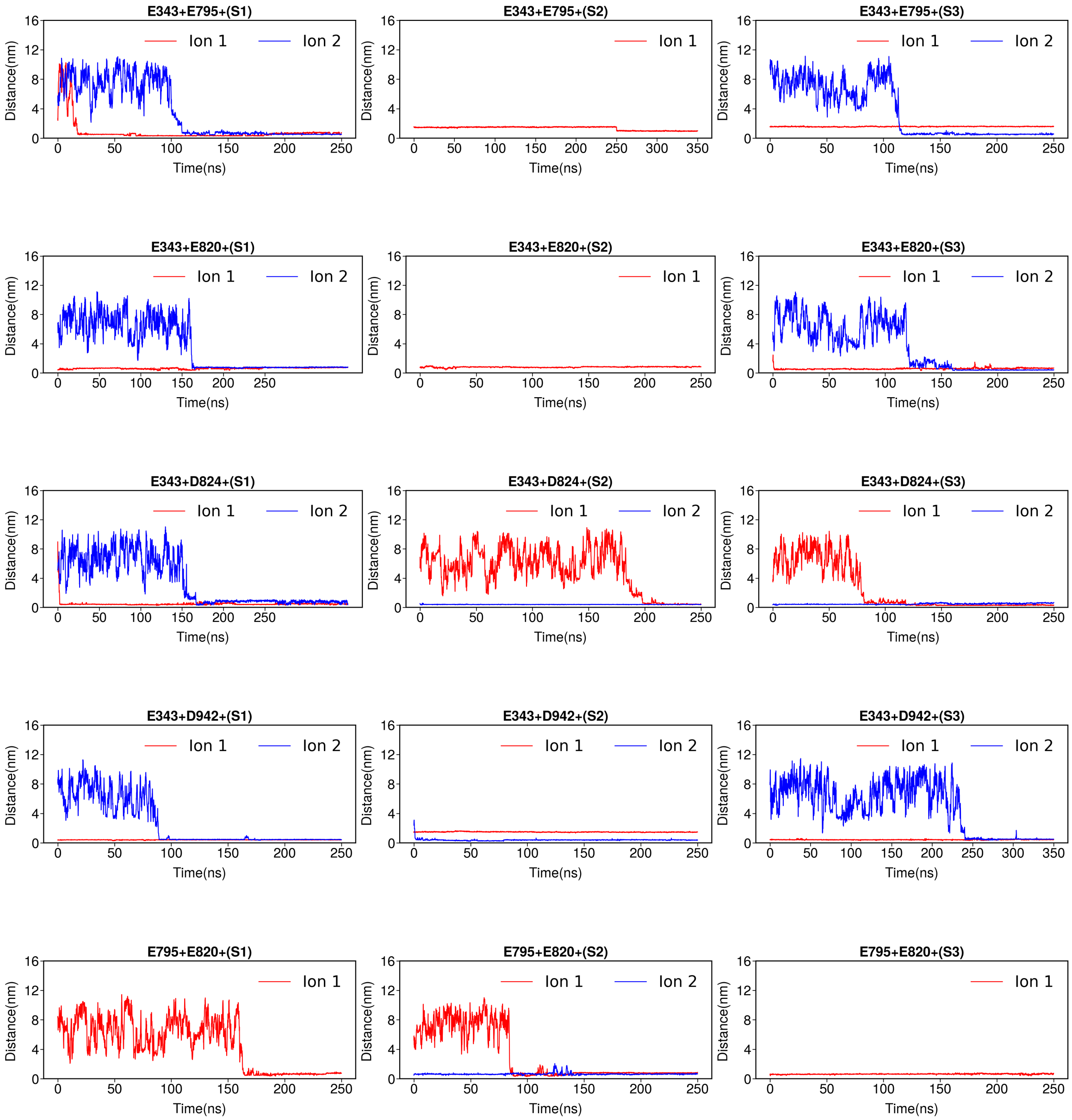

Figure SI1: Traces of K^+ binding for all simulations. Traces for three copies (S1, S2 and S3) for each protonation state are shown. The distance between the K^+ ion and residue E820 is shown in all panels. Except for the case of E795+D824+D942+, where a bound ion gets displaced by a different ion, ions once bound do not leave the binding site. Note that ions sometime bind in the equilibration phase (first 50 ns) of the simulations, and these traces are not shown in the figure.

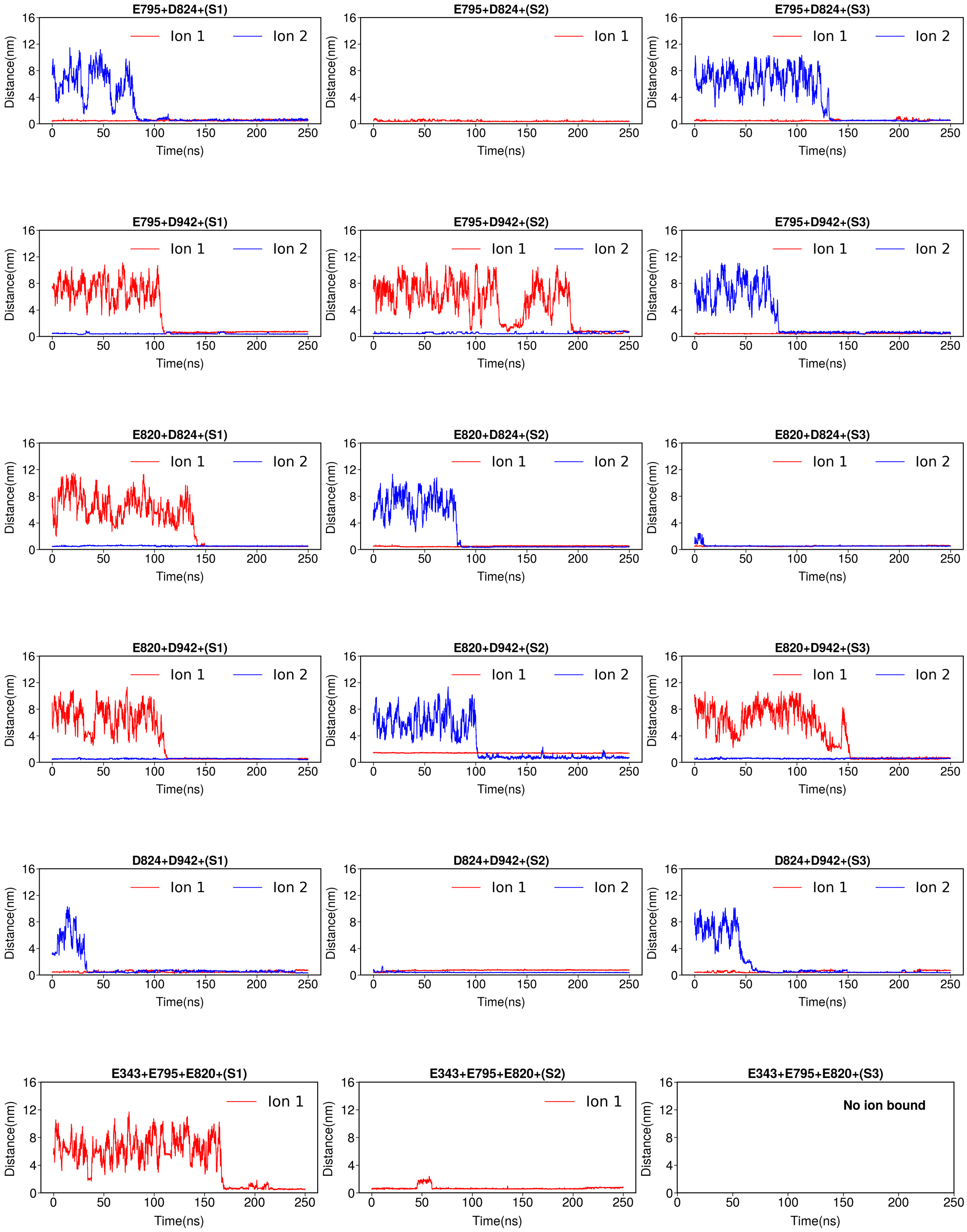
E795+D824+(S1)
Ion 1
Ion 2
E795+D824+(S2)
Ion 1
E795+D824+(S3)
Ion 1
Ion 2
E795+D942+(S1)
Ion 1
Ion 2
E795+D942+(S2)
Ion 1
Ion 2
E795+D942+(S3)
Ion 1
Ion 2
E820+D824+(S1)
Ion 1
Ion 2
E820+D824+(S2)
Ion 1
Ion 2
E820+D824+(S3)
Ion 1
Ion 2
E820+D942+(S1)
Ion 1
Ion 2
E820+D942+(S2)
Ion 1
Ion 2
E820+D942+(S3)
Ion 1
Ion 2
D824+D942+(S1)
Ion 1
Ion 2
D824+D942+(S2)
Ion 1
Ion 2
D824+D942+(S3)
Ion 1
Ion 2
E343+E795+E820+(S1)
Ion 1
E343+E795+E820+(S2)
Ion 1
E343+E795+E820+(S3)
No ion bound
Distance(nm)
Time(ns)
0
4
8
12
16
0
50
100
150
200
250

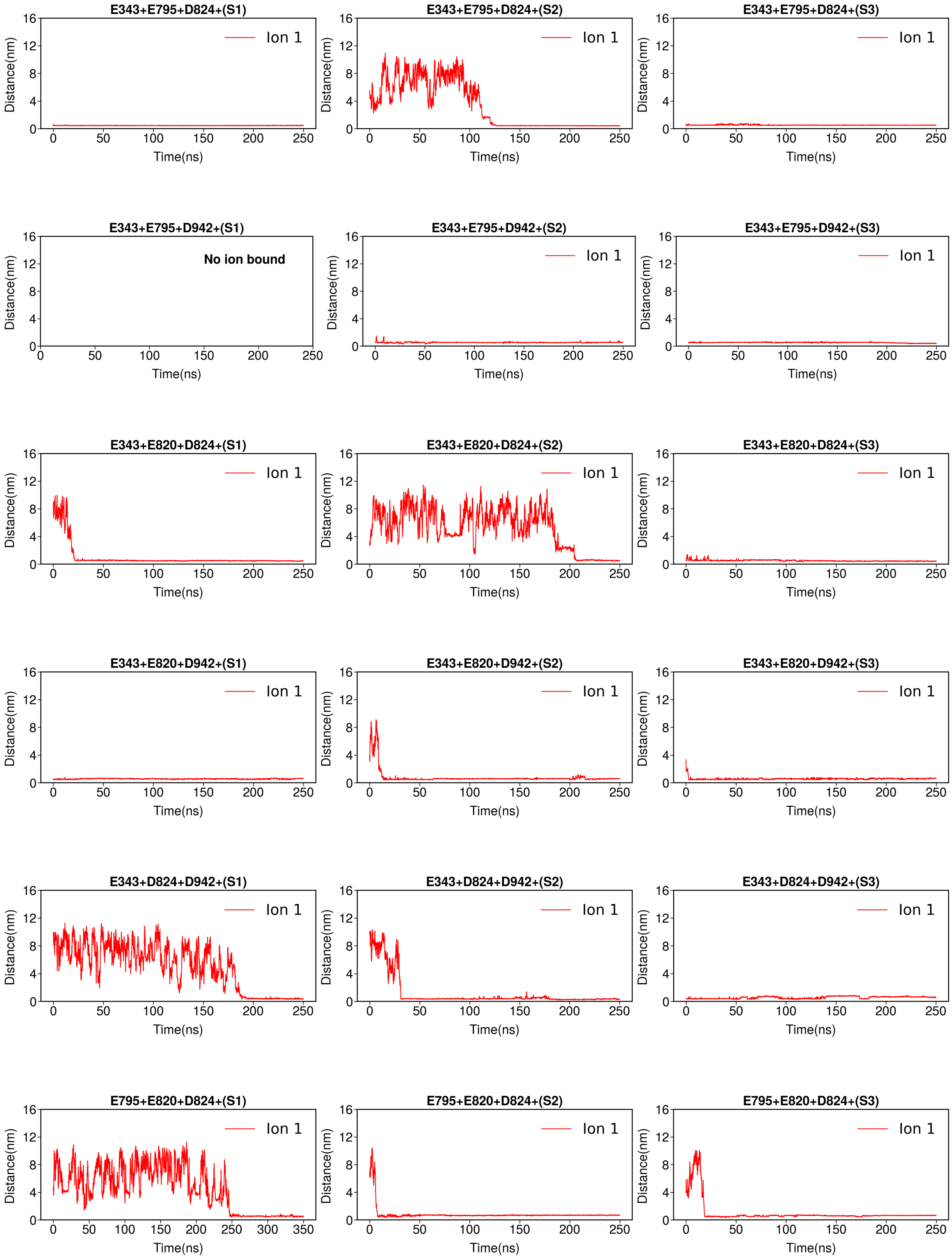
E343+E795+D824+(S1)
E343+E795+D824+(S2)
E343+E795+D824+(S3)
E343+E795+D942+(S1)
No ion bound
E343+E795+D942+(S2)
E343+E795+D942+(S3)
E343+E820+D824+(S1)
E343+E820+D824+(S2)
E343+E820+D824+(S3)
E343+E820+D942+(S1)
E343+E820+D942+(S2)
E343+E820+D942+(S3)
E343+D824+D942+(S1)
E343+D824+D942+(S2)
E343+D824+D942+(S3)
E795+E820+D824+(S1)
E795+E820+D824+(S2)
E795+E820+D824+(S3)
Ion 1
Distance(nm)
Time(ns)

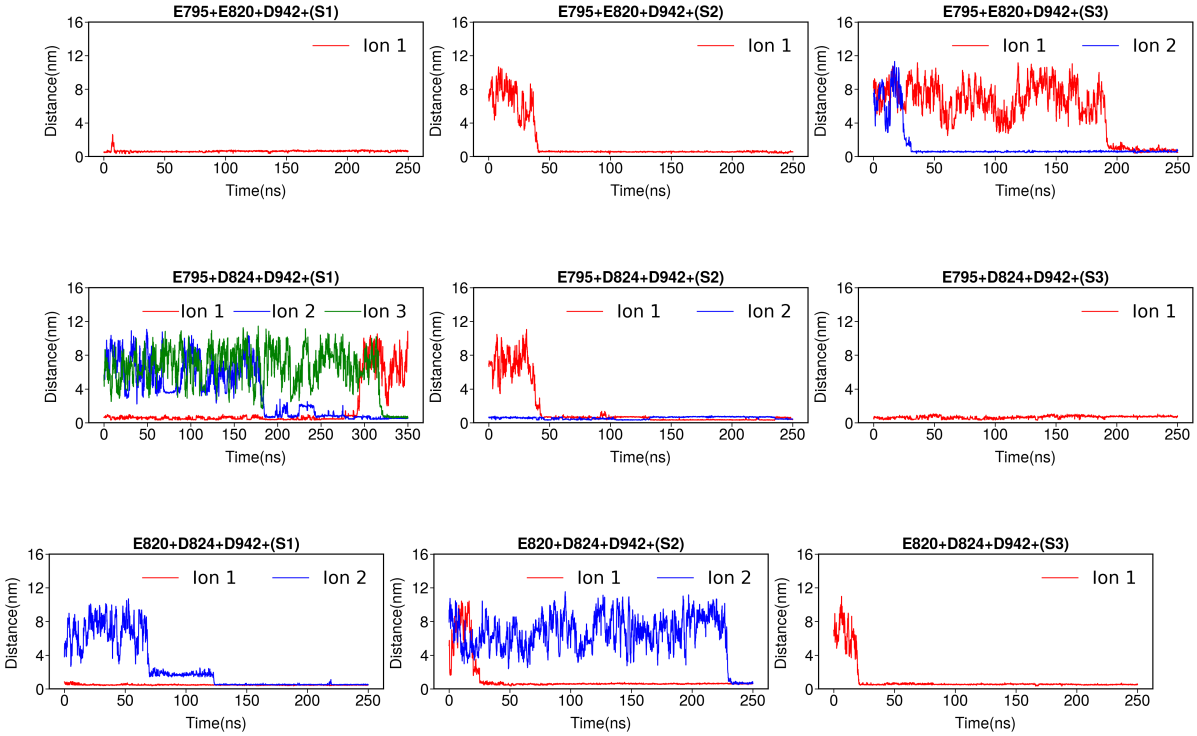
E795+E820+D942+(S1)
E795+E820+D942+(S2)
E795+E820+D942+(S3)
E795+D824+D942+(S1)
E795+D824+D942+(S2)
E795+D824+D942+(S3)
E820+D824+D942+(S1)
E820+D824+D942+(S2)
E820+D824+D942+(S3)
Ion 1
Ion 2
Ion 3
Distance(nm)
Time(ns)

Figure SI2: Radial distribution functions between pairs of acidic residues for the protonation states reported in Figure 3. The corresponding Pearson correlation coefficients are shown in parentheses. Residues which are more likely to exchange protons as predicted by the pKa correlation analysis in Figure 3, are also spatially adjacent as predicted by the radial distribution functions.

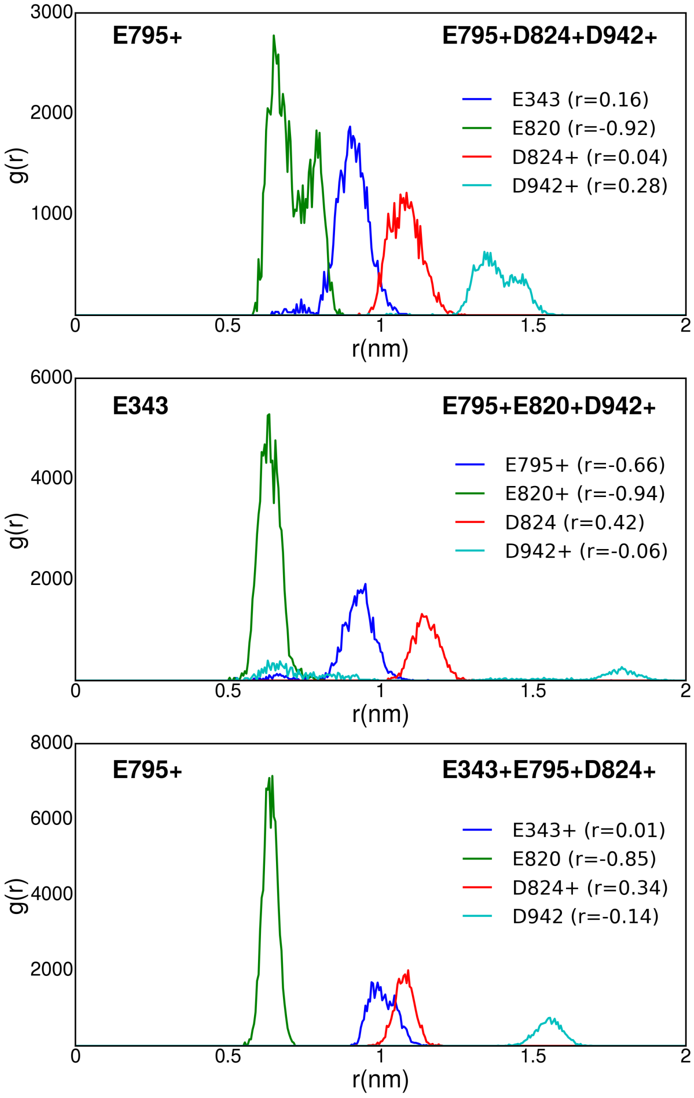

Figure SI3: pKa correlation analysis for systems equivalent to those in Figure 3, but with one less proton in the ion-binding site. The residues pairs predicted to exchange protons from the pKa correlation analysis are identical for systems with 2 or 3 protons. The correlation coefficient between E820 and E343 in the middle panels is smaller for the system with two protons, because both the residues are negatively charged, and are likely to repel.

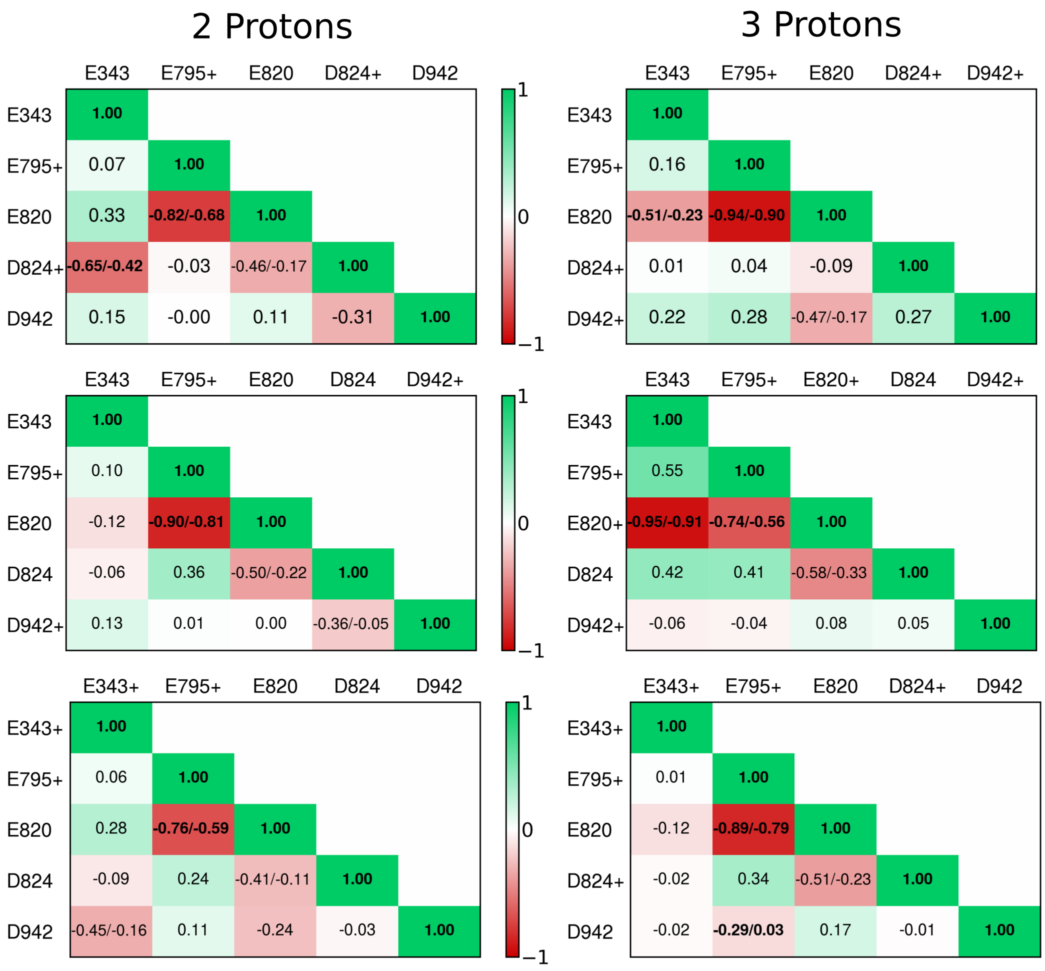

Figure SI4: Overlap between windows used for the PMF calculations for the protonation state E795+D824+D942+.

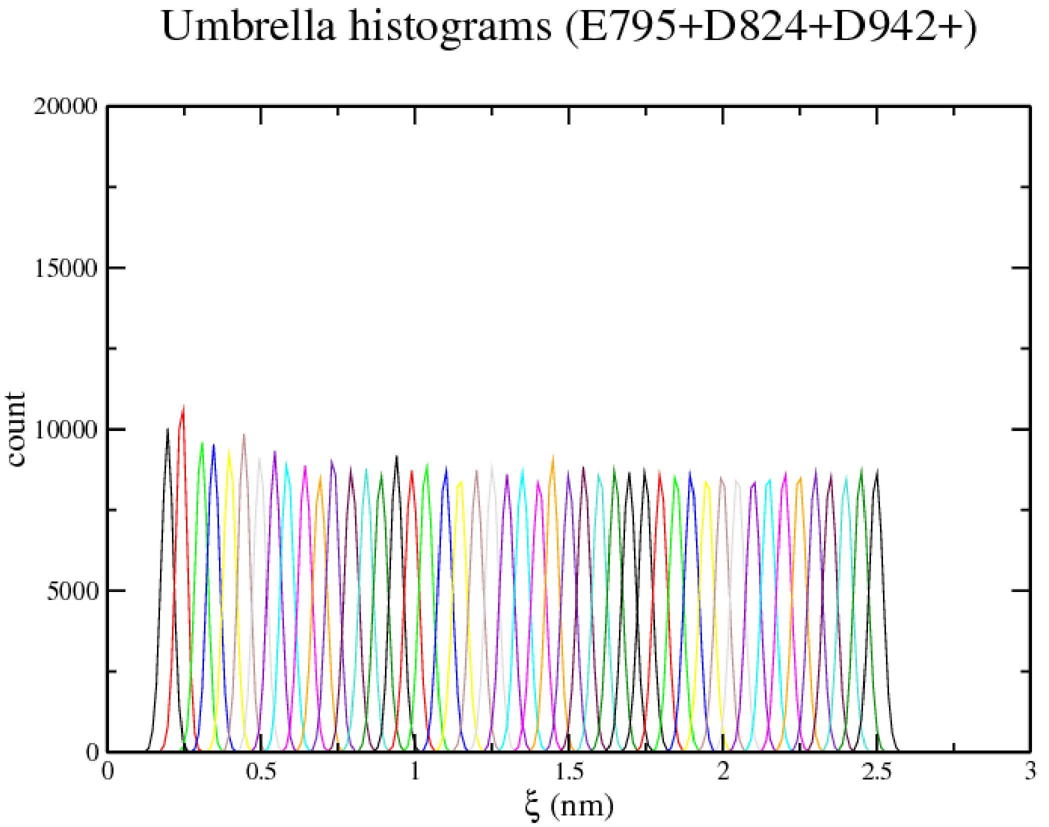

Supplement: Supplementary file 1 — Supplementary Information [file 41598_2018_30885_MOESM1_ESM.pdf]
